# Current perspectives of nanoparticles in medical and dental biomaterials

**DOI:** 10.7555/JBR.26.20120027

**Published:** 2012-05-22

**Authors:** Ibrahim Mohamed Hamouda

**Affiliations:** Department of Dental Biomaterials and Restorative Dentistry, Faculty of Dentistry, Mansoura University, Mansoura, Dakahleya 35516, Egypt.

**Keywords:** nanoparticles, nano-medicine, nano-biomaterials, nanotechnology, antibacterial agent

## Abstract

Nanotechnology is gaining tremendous impetus due to its capability of modulating metals into their nanosize, which drastically changes the chemical, physical and optical properties of metals. Nanoparticles have been introduced as materials with good potential to be extensively used in biological and medical applications. Nanoparticles are clusters of atoms in the size range of 1-100 nm. Inorganic nanoparticles and their nano-composites are applied as good antibacterial agents. Due to the outbreak of infectious diseases caused by different pathogenic bacteria and the development of antibiotic resistance, pharmaceutical companies and researchers are searching for new antibacterial agents. The metallic nanoparticles are the most promising as they show good antibacterial properties due to their large surface area to volume ratios, which draw growing interest from researchers due to increasing microbial resistance against metal ions, antibiotics and the development of resistant strains. Metallic nanoparticles can be used as effective growth inhibitors in various microorganisms and thereby are applicable to diverse medical devices. Nanotechnology discloses the use of elemental nanoparticles as active antibacterial ingredient for dental materials. In dentistry, both restorative materials and oral bacteria are believed to be responsible for restoration failure. Secondary caries is found to be the main reason to restoration failure. Secondary caries is primarily caused by invasion of plaque bacteria (acid-producing bacteria) such as *Streptococcus mutans* and *lactobacilli* in the presence of fermentable carbohydrates. To make long-lasting restorations, antibacterial materials should be made. The potential of nanoparticles to control the formation of biofilms within the oral cavity is also coming under increasing scrutiny. Possible uses of nanoparticles as topically applied agents within dental materials and the application of nanoparticles in the control of oral infections are also reviewed.

## INTRODUCTION

“Nano” is a Greek word synonymous to dwarf, meaning extremely small. The field of nanotechnology is one of the most popular areas for current research and development in basically all disciplines. Some evidence proved the safety of the application of nano-structured materials[1]. Bionanotechnology has emerged as biotechnology and nanotechnology become integrated for development of biosynthetic and environment-friendly technology and synthesis of nanomaterials.

Recently, with increasing public knowledge about health care in the world, people are increasingly concerned about the emergence of possible subsequent diseases caused by new technologies including nanotechnology and application of nano-materials. The development of a reliable and green chemistry process for biogenic synthesis of nanomaterial is an important aspect of current nanotechnology research[2]. Nanotechnology refers broadly to a field of applied science and technology whose unifying theme is the control of matter on the atomic and molecular scale. Nano science involves the study of materials on the nano-scale level between approximately 1 and 100 nm. Metal-microbe interactions have an important role in several biotechnological applications including the fields of biomineralization, bioleaching, and microbial corrosion[3],[4]. Inorganic and metallic-based nano-structured materials have created a new interesting field in all sciences for continuous investigations due to their undeniably unique properties. Their applications have already led to the development of new practical productions[5]. In the past few years, nano-structured materials have been receiving considerable attention as a result of their unique physical and chemical properties, biological properties, and functionality due to their nano-scale size, and have elicited much interest and important applications in optics and biomedicine[6],[7].

An important aspect of nanotechnology is the development of toxicity-free synthesis of metal nanoparticles, which is a great challenge. The secrets discovered from nature have led to the development of biomimetic approaches to the growth of advanced nano-materials[8]. The interaction of nanoparticles with biomolecules and microorganisms is an expanding field of research. Many investigations have focused on their bactericidal effect and applications in plastics and health[9]. In recent years, a rapid increase in microbes that are resistant to conventionally-used antibiotics has been observed[10]. With the emergence and increase of microbial organisms resistant to multiple antibiotics, and the continuing emphasis on healthcare costs, many researchers have tried to develop new and effective antimicrobial reagents free of resistance and cost. Such problems and needs have led to the resurgence in the use of nano-sized antiseptics that may be linked to broad-spectrum activity and far lower propensity to induce microbial resistance than antibiotics[11].

Recent studies have demonstrated that specially formulated metal oxide nanoparticles have good antibacterial activity[12], and antimicrobial formulations comprised of nanoparticles could be effective bactericidal materials[13],[14]. Antimicrobial materials fight bacteria and delay, reduce or avoid the formation of biofilms on the materials. There are different strategies to accomplish this. Generally, antimicrobial properties of (bio) materials may be accomplished by introducing agents such as silver[15] or one or more antibiotics into the materials. Microbes are subsequently killed following contact with the materials or through leaching of the antimicrobial agents into the body environment[16].

Resistant strains of microorganisms will fail to develop if we apply nanoparticle-based formulations in their culture media. In laboratory tests with nanoparticles, the bacteria, viruses, and fungi were killed within minutes of contact[7]. The size of metallic nanoparticles ensures that a significantly large surface area of the particles is in contact with the bacterial effluent. Considering a hypothetical case with spherical particles of uniform size, a reduction in the particle size from ∼10 µm to 10 nm will increase the contact surface area by 10^9^. Such a large contact surface is expected to enhance the extent of bacterial elimination. However, smallness in itself is not the goal. Synthesis and characterization of nano-scaled materials in terms of novel physicochemical properties is of great intervest in the formulation of bactericidal materials[17].

Because of huge surface free energy, nanoparticles bond strongly to other materials or to each other (agglomeration). These effects may be exploited in bulk applications of nanoparticles[18]. In restorative dentistry, there has also been a growing interest in using nanoparticles to improve properties of dental restoratives[19]. This review paper is concerned with the most commonly used inorganic nano-structured materials with good povtential of anti-microbial activity and the effect of nano-composites on the properties of dental biomaterials.

## NANO-STRUCTURED MATERIALS AND THEIR APPLICATIONS

Nanotechnology includes the integration of these nano-scale structures into larger material components and systems, keeping the control and construction of new and improved materials at the nanoscale. In addition, these nano-materials also present different interesting morphologies such as spheres, tubes, rods and prisms. Inorganic nanoparticles including the ones based on metal oxides (zinc oxide, iron oxide, titanium dioxide and cerium oxide), metals (gold, silver and iron, copper, and magnesium), and quantum dots (cadmium sulfide and cadmium selenide)[20]-[23]. Additionally, silicon dioxide and aluminum oxide nanoparticles have been used[23]. Alginate nano-materials can also be used as antimicrobial agents[24]. Mixtures of different phases are also manufactured. Silver has come up but silver nanoparticles have been proved to be most effective as they have good antimicrobial efficacy against bacteria, viruses, and other eukaryotic microorganisms[20].

As a result of their small size, nanoparticles may offer other advantages to the biomedical field through improved biocompatibility[25]. Additionally, it appears that bacteria are far less likely to acquire resistance against metal nanoparticles than other conventional and narrow-spectrum antibiotics. This is thought to occur because metals may act on a broad range of microbial targets, and many mutations should occur for microorganisms to resist their antimicrobial activity. Shape may also affect the activity of nanoparticles[26].

Other metals have been used for centuries as antimicrobial agents. Copper, gold, titanium, and zinc have attracted particular attention, with each having different properties and spectra of antimicrobial activity[27],[28]. The broad antibacterial activity of nano-silver reduces patient infection, dependence on antibiotic use, and associated costs. There is room for improvement in stabilizing and prolonging the antibacterial effects of nano-silver coatings for medical applications to prevent infection and inflammation. Finally, with the widespread adoption of nano-silver, several concerns about toxicity remain and need to be addressed[29]. The use of silver has been severely limited by the toxicity of silver ions to humans. However, nanotechnology has facilitated the production of smaller silver particles with increasing large surface area-to-volume ratios, greater efficacy against bacteria[30] and, most importantly, lower toxicity to humans[31]. The mechanisms underlying the impressive biological properties of nano-silver are still not understood and this is a priority for future research *in vivo*[29].

Both silver and titanium particles were introduced into dental composites, to introduce antimicrobial properties and enhance biocompatibility of the composites[25],[32],[33]. Nano-silver, comprising silver nanoparticles, is attracting interest for a range of biomedical applications owing to its potent antibacterial activity. It has recently been demonstrated that nano-silver has useful anti-inflammatory effects. Silver nanoparticles, or nano-silver, are clusters of silver atoms that range in diameter from 1 to 100 nm and are attracting interest as antibacterial and antimicrobial agents for applications in medicine[29]. Moreover, nano-silver exhibits remarkable biological properties, such as antiviral activities[34],[35]. The action of nano-silver depends on the inhibition of fusion or entry of the virus into the host cell, since blocking HIV entry into its target cells can lead to the suppression of viral infectivity, replication, and cytotoxicity induced by virus-cell interaction[36]. In addition to fusion inhibitors, virucidal agents are also urgently needed for HIV/AIDS prevention because they directly inactivate virus particles (virions), thus preventing the completion of the virus replication cycle. Virucidal agents differ from virustatic drugs in that they act directly and rapidly by lysing viral membranes on contact or by binding to virus coat proteins[37]. Silver nanoparticles are effective virucides as they inactivate HIV particles in a short period of time, exerting their activity at an early stage of viral replication (entry or fusion) and at post-entry stages[38].

The antimicrobial property of silver is related to the amount of silver and the released rate of silver. Silver in its metallic state is inert, but it reacts with the moisture in the skin and the fluid of the wound and gets ionized. The ionized silver is highly reactive, as it binds to tissue proteins and brings structural changes in the bacterial cell wall and nuclear membrane, leading to cell distortion and death[39]. The possible mechanisms underlying the action of metallic silver, silver ions and silver nanoparticles have been proposed according to the morphological and structural changes found in the bacterial cells[34]. The effect of silver ions on bacteria can be observed by the structural and morphological changes. It is believed that silver ions interact with the three main components of bacterial cells to produce the bactericidal effect: the peptidoglycan cell wall[40],[41] and plasma membrane[42], bacterial DNA[43], and bacterial proteins, especially enzymes involved in vital cellular processes such as the electron transport chain[40]. It is reported that silver ions cause the lysis of bacterial cells[33].

Furthermore, it is reported that heavy metals react with proteins by getting attached with the thiol group and the proteins get inactivated[33],[44]. Silver nanoparticles show efficient antimicrobial property compared to other salts due to their extremely large surface area, which provides better contact with microorganisms[33]. Studies have shown that the positive charge on the metal ion is critical for the antimicrobial activity of sliver nanoparticles, allowing the electrostatic interaction between negatively charged bacterial cell membranes and positively charged nanoparticles[25]. Panacek *et al*.[45] and Pal *et al*.[26] reported a one step protocol for the synthesis of silver colloid nanoparticles. They found high antimicrobial and bactericidal activity of silver nanoparticles on Gram-positive and Gram-negative bacteria including multi-resistant strains such as methicillin resistant *Streptococcus aureus*. The antibacterial activity of silver nanoparticles was found to be size-dependent, and the nanoparticles of 25 nm possessed the highest antibacterial activity. However, the nanoparticles were toxic to bacterial cells at a low concentration of 1.69 µg/mL Ag.

A comparative study of nano-silver, silver nitrate and silver chloride revealed that nano-silver particles showed higher antibacterial potency than free silver ions[46]. Silver has been in use since time immemorial in the form of metallic silver, silver nitrate, and silver sulfadiazine for the treatment of burns, wounds and several bacterial infections[34]. This suggests that nanosilver has intrinsic antibacterial properties that do not depend on the elution of Ag^+^. Nano-silver exhibits antibacterial effects against a large number of bacterial species. Nano-silver contributes to the broadspectrum antibacterial activity. Furthermore, bacterial resistance to elemental silver is extremely rare[34],[47],[48]. Silver nanoparticles were greatly influenced by the concentration of AgNO_3_ solution. Typically, silver nanoparticles were well dispersed on the vanadium oxide nanotubes with the size range from 3 to 10 nm. The corresponding antibacterial tests demonstrated the synthesized vanadium oxide nanotubes exhibited strong antibacterial activity against Escherichia coli[49].

Little information is available regarding the antibacterial effects of silver ions and silver nanoparticles under anaerobic conditions. The antibacterial activity of silver-zeolite was demonstrated as it inhibited the growth of the tested bacteria under anaerobic conditions. These results suggested that silver-zeolite may be a useful vehicle to provide antibacterial activity to dental materials, even under anaerobic conditions such as deep in the periodontal pocket. Silver zeolite has been evaluated against a range of obligate and facultative anaerobic oral species. Gram-negative species (*Porphyromonas gingivalis*, *Prevotella intermedia*, and *Aggregatibacter actinomycetemcomitans*) were shown to be more susceptible than Gram-positive species (*S. mutans*, *S. sanguinis*, and *Actinomyces viscosus*)[50].

With respect to nanoparticles, the antimicrobial properties of copper have also received the most attention. Both of these have been coated onto or incorporated into various materials[51]. An inverse relationship between nanoparticle size and antimicrobial activity has been demonstrated: nanoparticles in the size range of 1-10 nm have the greatest bactericidal activity against bacteria[33],[52]. Copper oxide is cheaper than silver, easily mixed with polymers, and relatively stable in terms of both chemical and physical properties. Copper oxide nanoparticles have been characterized physically and chemically and investigated with respect to their potential antimicrobial applications. It was found that nano-scaled CuO generated by thermal plasma technology was demonstrated to possess a particle size of 20 to 95 nm, with a mean surface area of 15.7 m^2^/g. CuO nanoparticles in suspension were demonstrated to show activity against a range of bacterial pathogens. However, compared with CuO, silver nanoparticles showed greater bactericidal activity. Like silver, studies of CuO nanoparticles incorporated into polymers suggest that the release of ions may be required for optimum killing[53].

### Nanoparticles in dental applications

Most dental treatments become necessary when pathogenic germs colonize the dentine and enamel, the marginal gaps between the dentine and enamel and dental restorations, restoration and prosthetic materials as well as the neighboring soft tissue[54]. In particular, the bacteria such as *S. mutans* and *S. lactobacilli* produce acids, which cause extensive dental caries and severe damage of hard tissues. Thus, when a root canal is filled with known inert filling materials, germs that remain in the canal will gradually cause an inflammatory process after filling, which makes a renewed treatment necessary or leads to entire loss of teeth. Antimicrobial dental materials are frequently used to preclude these destructive treatments[51]. The antimicrobial action is most often achieved by adding active antimicrobial ingredients to the dental material. A restorative material that possesses antibacterial properties and inhibits bacterial growth around the restoration would be desirable. As a means of reducing bacterial and fungal adhesion to dental materials and devices, silver nanoparticles are being investigated for a range of possible applications, for example, incorporation into denture materials and orthodontic adhesives[55].

Dental materials with antimicrobial activity such as filling materials, cements, sealants, materials for temporary restorations, coating materials and adhesives have emerged[56]. A problem is that the physical and chemical properties of the dental material, such as its mechanical properties or the hardening behavior must not be affected by the addition of the active ingredients. The release of active ingredients in an effective quantity and over an extended and clinically relevant time span must also be ensured[54]. The incorporation of silver nanoparticles into bonding adhesives was successful on both physical and antimicrobial levels[57].

Silver ions have been considered as antibacterial components in dental resin composites[58]. The modified tissue conditioner combined with silver nanoparticles displayed antimicrobial properties against *S. aureus, S. mutans* and *C. albicans* incorporated after a 24-h or 72-h incubation[59]. Light cured flowable composite resin materials can be made to function as an antimicrobial product by the addition of silver hydrosol. The silver hydrosol can be released (at a steady rate over time) from the resin composite matrix to reduce the incidence of tooth decay[51]. Silver colloid nanoparticles were added to polymeric adhesive to improve the efficiency of electrical conduction[60]. Recently, quaternary ammonium poly(ethylene imine) (QA-PEI) nanoparticles were developed for additional antibacterial activity of restorative composite resins. QA-PEI nanoparticles completely inhibited the growth of *S. mutans*, and their antibacterial activity lasted at least 3 months[61].

The electrical and flexural properties of silver nanvoparticles-filled epoxy composites were improved[62]. The incorporation of silver nitrate and silver nanoparticles (AgNPs) significantly reduced the adhesion of *C. albicans* to the acrylic resin surface, suggesting that AgNPs-combined denture base materials may be a potential approach to prevent denture stomatitis[63]. There are a number of factors that need to be considered in silver nanoparticles-filled epoxy composites such as filler concentration, filler shape and size, and filler composition to modify the properties of metal filled polymer composites[62].

Silver-zinc antimicrobial zeolites were added in low percentages to polymethyl methacrylate[64]. It can also be used as a valuable alternative to reduce microbial contamination of tissue conditioners, acrylic resin denture bases, and acrylic base plates of removable orthodontic appliances. Zeolites are aluminum silicate crystalline structures. Addition of 2.5% of zeolites to the materials resulted in decreased flexural strength and impact strength[65],[66]. Silver zeolite nanoparticles have been incorporated into mouth rinses and toothpastes[65]. Now, powdered zinc citrate or acetate has been incorporated to control the formation of dental plaque. Powdered titanium dioxide is also commonly used as a whitener in toothpastes[27],[28]. Additionally, nanoparticles can be used effectively in other materials including hydrogels[67].

A variety of permanent dental cements can be impregnated with silver hydrosol including epoxy resin cements, glass ionomer and resin modified glass ionomer cements (used in permanent cement crowns and bridge work). Any number of commonly used permanent dental cements can also be readily combined with the silver hydrosol solution. By adding the silver hydrosol to these cements, one is able to provide a continuous dynamic antimicrobial bacteriostatic environment capable of reducing bacterial bioburden and thus postoperative inflammation, infection and sensitivity, which are particularly important with vital teeth[54]. Novel poly quaternary ammonium salt-containing antibacterial glass-ionomer cement was developed. All the poly quaternary ammonium salt-containing cements showed a significant antibacterial activity, accompanying with a reduction of initial compressive strength. In addition, it was concluded that the experimental cement is a clinically attractive dental restorative due to its high mechanical strength and antibacterial function[68].

Alginate impression powders can be mixed with water that contains silver hydrosol to create an impression material that has antimicrobial activity. This will reduce microbial cross contamination by bacteria, yeasts, other fungi and viruses to the stone model from the infected impression[67]. Antimicrobial root canal sealer/cements with the addition of dilute silver hydrosol are useful in permanent obturation of the root canal following removal of the infected pulp and placement of medicaments.

Scanning electron microscope (SEM) observation of the dispersibility of silver-zirconium phosphate (SZP) nano-inorganic antimicrobial agent in silicone denture soft lining materials indicated that the inorganic nano-granules were well distributed in silicone substrate. Element analysis demonstrated the even distribution of zirconium and silver, which confirmed that there were not obvious nano-agglomerates, and in turn verified the excellent dispersibility of SZP in tested silicone[69]. Transmission electron microscopy (TEM) analysis and atomic adsorption spectroscopy revealed that silver nanoparticles are compatible with the acrylic formulation and remain well-dispersed in the final material. Silver nanoparticles have no detrimental effect on the photopolymerization kinetics and the incorporation of nanoparticles was found to reduce the gloss of ultraviolet-cured coatings[70].

Research efforts are currently directed towards eliminating or reducing infection of medical devices. Strategies to prevent biofilm formation include physiochemical modification of the biomaterial surface to create anti-adhesive surfaces, incorporation of antimicrobial agents into medical device polymers, mechanical design alternatives, and release of antibiotics[71]. In this context, zinc oxide nanoparticles have undergone *in vitro* testing in biofilm culture test systems. Zinc oxide nanoparticles blended into a variety of composites were shown to significantly inhibit the growth of *S. sobrinus* biofilm over a three-day test period[27]. Kishen *et al*.[72] demonstrated a reduction in the number of *E. faecalis* adhered to the dentine on the surface of the root canal treated with cationic antibacterial nano-particulates such as zinc oxide alone or the combination of zinc oxide and chitosan nano-particulates. In theory, such surface treatment could prevent bacterial recolonization and biofilm formation *in vivo*.

Particles of a nano- and micro-size based upon the element silicon for the rapid delivery of antimicrobial and anti-adhesive capabilities to the desired site within the oral cavity have received much attention[73]. Some companies have used silica (silicon dioxide, SiO_2_) with a particle size within the definition of nanoparticles in toothpastes for many years, and some are now actively seeking new directions in this area through the use of porous silicon/nano-crystalline silicon technology to carry and deliver antimicrobials such as triclosan[27].

The mechanical properties of SiO_2_ nanoparticles were improved even at low filler content[74]. Nanoparticle filled dental composites may show an enhanced fracture toughness and adhesion to tooth tissue[75]. The use of silica nanoparticles to polish the tooth surface may help protect against damage caused by cariogenic bacteria, presumably because the bacteria can be removed more easily. This has been investigated on human teeth *in vivo*[76]. Modified surfaces were shown to reduce the attachment and growth of *C. albicans*, with the greatest effect observed with 7- and 14-nm particles. Such effects could possibly be attributed to surface topography or slow dissolution of the bound silica. Such treatment has the advantages of being non-toxic, simple to apply, and adaptable to three-dimensional surfaces[27].

Bioactive glasses of the SiO_2_-Na_2_O-CaO-P_2_O_5_ system have been shown to possess antimicrobial activity through the release of ionic alkaline species over time and are under consideration as dentine disinfectants to offer an alternative to calcium hydroxide. Those in the form of amorphous nanoparticles with a size of 20 to 60 nm may show an advantage over micron-sized material, because the decrease in glass particle size should increase the ionic release into suspension and enhance antimicrobial efficacy. Antimicrobial activity was assessed against *E. faecalis*, a pathogen often isolated from root canal infections. The killing efficacy of the nano-sized particles was also significantly higher[77].

Therefore, nanoparticles might improve the mechanical properties such as wear resistance and surface hardness of dental restorative materials[78]. The major difference between nano-metric and micrometric particles is that nanoparticles have significantly larger specific surface area, which greatly facilitates the transfer of load from polymer matrix to nanoparticles[79]-[81]. As a result, nanoparticle-reinforced hybrid system exhibits higher stiffness and better resistance to wear[79].

Glass ionomer-containing (3% and 5%, *W*/*W*) TiO_2_ nanoparticles showed improved fracture toughness, flexural strength and compressive strength compared to the unmodified glass ionomer. However, a decrease in mechanical properties was found for glass ionomer-containing (7%, *W*/*W*) TiO_2_ nanoparticles. Glass ionomer-containing (5% and 7%, *W*/*W*) TiO_2_ nanoparticles compromised the surface micro-hardness. Setting time of glass ionomer-containing TiO_2_ nanoparticles is accepted and meets the requirement of water-based cements. The addition of TiO_2_ nanoparticles to the conventional glass ionomer did not compromise its bond strength with dentine or fluoride release of the glass ionomer. Glass ionomer-containing TiO_2_ nanoparticles possessed the most potent antibacterial activity against *S. mutans* compared to the unmodified glass ionomer[82].

## CONCLUSION

Nanoparticles have come up as one of the most effective antibacterial agents due to their large surface area to volume ratios. They can be used as effective growth inhibitors of various microorganisms. Furthermore, nanomaterials can be modified of achieve better efficiency and to facilitate their applications in different fields such as biomaterials and medicine. The long-term antibacterial, physical and clinical effects of nanoparticles on dental and medical biomaterials should be investigated in future studies.
